# Prehospital analgesia using nasal administration of S-ketamine – a case series

**DOI:** 10.1186/1757-7241-21-38

**Published:** 2013-05-14

**Authors:** Joakim Johansson, Jonas Sjöberg, Marie Nordgren, Erik Sandström, Folke Sjöberg, Henrik Zetterström

**Affiliations:** 1 The research and development unit, Jämtland county council, Östersund, Sweden; 2 Department of Anaesthesiology and Intensive Care, Östersund hospital, Östersund SE 83183, Sweden; 3 Department of Anaesthesia and Intensive Care, Linköping University Hospital, Linköping, Sweden; 4 Department of Clinical and Experimental Medicine, Faculty of Health Sciences, Linköping University, Linköping, Sweden; 5 Department of Anaesthesiology and Intensive Care, Uppsala University, Uppsala, Sweden

**Keywords:** Analgesia, Drug administration, Intranasal, Emergency, Ketamine, Prehospital, S-ketamine, Trauma

## Abstract

Pain is a problem that often has to be addressed in the prehospital setting. The delivery of analgesia may sometimes prove challenging due to problems establishing intravenous access or a harsh winter environment. To solve the problem of intravenous access, intranasal administration of drugs is used in some settings. In cases where vascular access was foreseen or proved hard to establish (one or two missed attempts) on the scene of the accident we use nasally administered S-Ketamine for prehospital analgesia. Here we describe the use of nasally administered S-Ketamine in 9 cases. The doses used were in the range of 0,45-1,25 mg/kg. 8 patients were treated in outdoor winter-conditions in Sweden. 1 patient was treated indoor. VAS-score decreased from a median of 10 (interquartile range 8-10) to 3 (interquartile range 2-4). Nasally administered S-Ketamine offers a possible last resource to be used in cases where establishing vascular access is difficult or impossible. Side-effects in these 9 cases were few and non serious. Nasally administered drugs offer a needleless approach that is advantageous for the patient as well as for health personnel in especially challenging selected cases. Nasal as opposed to intravenous analgesia may reduce the time spent on the scene of the accident and most likely reduces the need to expose the patient to the environment in especially challenging cases of prehospital analgesia. Nasal administration of S-ketamine is off label and as such we only use it as a last resource and propose that the effect and safety of the treatment should be further studied.

## Background

The county of Jämtland and Härjedalen is a sparsely populated area in the central/west part of Sweden close to the mountain-range that borders to Norway. Its area is 49000 km^2^ which is slightly greater than that of Denmark. The only town, Östersund, harbours the only hospital in the county and is situated 100–180 kilometers from the most popular mountain areas with heavy downhill and cross-country skiing activities and many ski resorts. Due to the long distances involved in prehospital care, the road ambulance service is complemented by an air ambulance, a Dauphine AS 365 N2. It is operated by a standard crew of three; a pilot, a navigator/technician/paramedic and an anesthesia nurse. The latter always has many years experience of prehospital and in-hospital anesthetic care. An anesthesiologist from the hospital is included as occasion requires. The largest ski resort, Åre, also features a ski patrolling anaesthesia nurse (MN) designated to provide prehospital care.

The standard prehospital analgesia is intravenous fentanyl or morphine but establishing intravenous access may be challenging in some cases. In all cases it is desirable to limit the rescue-time, even more so when temperatures are low and hypothermia is a risk. The nasal mucosa is richly vascularised and uptake to the blood of aerosolized drugs with certain characteristics is possible [1].

We have found one report indicating use of nasal ketamine in mountain rescue [2] and one previous case report [3].

This case-series describes experiences of using nasal S-ketamine for prehospital analgesia in nine especially challenging cases during 2 winter seasons 2010–1012.

## Materials and methods

This is a series of nine cases that were treated on the scene where the decision to use off-label nasal S-ketamine were taken by the responsible nurse/physician. Hence, we present a case-series and as such no informed consent *for treatment* was sought from the patients. The regional ethics review board was approached with an application but waived the need for an approval to publish the data. The reason was that since the goal of our treatment was to deliver analgesia and not to test the effect of the medicine, it was considered a case-series which is not dealt with by the ethics review board. The board gave a guiding statement though, regarding the publication of data, that with informed consent this would be ethical.

Informed consent *for publication* of data was achieved later from patients or in cases of minors from one of their parents.

The presented nasal administration of S-ketamine was considered only in cases of traumatic injuries when vascular access was foreseen or proven problematic (1 or 2 missed attempts). Patients with a suspected traumatic brain injury or facial trauma with nasal bleed were not considered. A total of 14 cases were treated during the two winter seasons 2010–2012, nine cases were evaluated in the case-series. The reasons for not being presented are other strong analgetics given in parallel (sublingual fentanyl or intravenous morphine after iv-access was achieved) or that patients could not be reached for an informed consent.

We consider this treatment a last resort and use it in a minority of patients. During two normal years our prehospital service handles approximately 700 traumatic cases that are brought in to Östersund hospital. The 14 treated cases hence constitute approximately 2% of trauma cases.

Preservative-free S-ketamine 25 mg/ml (Ketanest®, Pfizer, New York, NY, USA) was delivered by means of a so called MAD 300 (Mucosal Atomization Devise, LMA North America Inc., San Diego, Ca, USA) and a disposable plastic 5 ml-syringe with a luer-lock connection (BD, Franklin Lakes, NJ, USA). The first dose given was 0,5 mg/kg body weight and thereafter additional doses were given as needed. The total dose was limited to 1,0 mg/kg body weight in adults and 1,5 mg/kg body weight in children. We delivered no more than 0,5 ml per nostril at a time and waited at least 2 minutes before the next administration in the same nostril.

The patients were asked to score their pain on a scale from 1 (min) to 10 (max) before and five to ten minutes after the treatment. The treating nurse/physician paid attention to side-effects (hypersecretion, signs of vertigo or nausea).

Pain-score was considered an ordinal variable and testing for differences before and after analgesia was done with Wilcoxons matched pairs test. A p-value <0.05 was considered statistically significant. Statistica 10 (StatSoft® Inc, Tulsa, OK, USA) was used for analysis.

## Results

The characteristics of the patients, dose (mg/kg body weight), pain scoring and registered side-effects are presented in Table 1.

**Table 1:** Demographics, doses, pain-scores at the scene of accident, and type of injury

**Case #**	**Age**	**Sex**	**Weight**	**Dose mg/kg**	**Pain score before**	**Pain score after**	**Injury**
1	7	M	28	0,45	10	3	Fractured lower leg
2	19	F	60	0,83	10	4	Fractured clavicle
3	13	M	40	1,25	10	6	Fractured ulna
4	22	M	90	1,11	6	6	Fractured lower leg
5	14	M	52	0,96	7,5	1	Contusion leg
6	36	M	76	0,98	3	3	Fractured lower leg
7	7	M	35	1,1	9	2	Fractured femur
8	13	M	45	0,83	10	2	Fractured humerus
9	17	F	nd	nd	10	1	Knee distorsion
**Median**	**16**		**48,5**	**0,94**	**10(8–10)**	**3 (2–4)**	

Data for Pain-score are presented as median (25th–75th percentile).

**Figure 1:**
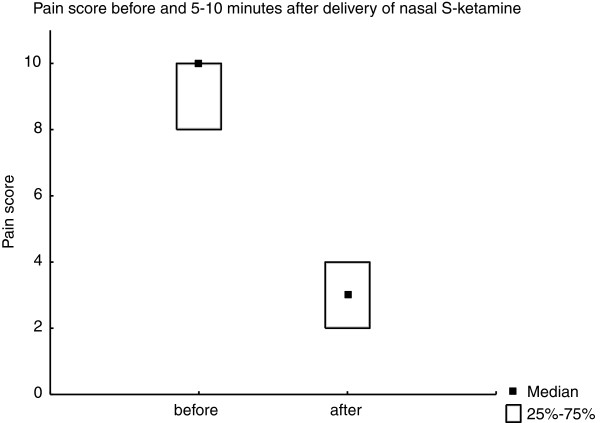
**Pain-score before and 5–10 minutes after analgesia with nasal S-ketamine.** Data presented as median (quartiles). There was a statistically significant difference in pain score (p=0.018).

Median pain-score before treatment at the scene of the accident was 10 (interquartile range 8–10) and median pain-score after was 3 (interquartile range 2–4) (Figure 1). The difference was significant (p=0.018).

## Discussion

When discussing pain treatment, the placebo effect must be considered. It is most likely reinforced by several factors in prehospital care, such as the ability of health personnel to act confident and reassuring, whether a relative or friend can accompany on the ride to the hospital, and so on. Other factors, beside pharmacological, that affect prehospital pain are the effectiveness of immobilization of the fractured limb [4] and the ability of health providers to move patients smoothly. In our case-series, the substantial drop in pain-score after S-ketamine administration strongly suggests an effect of the treatment. The contribution of placebo and other effects can’t be estimated in this case-series.

The median pain-score decreased from 10 to 3 (mean from 8,44 to 3,0). We believe that a pain-score of 3 is a reasonable target on the scene of an accident. Two patients were children (below 12 years old) and a total of five patients were 14 years old or younger. The nurses pointed out that nasal analgesia felt the most purposeful in young patients because of difficulties with vascular access and the non-cooperative state of some young patients in distress.

The need for a vascular access is not only related to analgesia but also to the risk of occult bleeding and hence to the possible need for resuscitation during transport or to give additional drugs that are not suited for nasal administration.

Patient # 4 scored 6–6 but received a dose similar to the other patients. It is unclear why the effect was insufficient. Large differences in plasma concentration after nasal ketamine administration have by earlier investigators been attributed to several factors: a variable amount of the drug swallowed [5] which may in turn depend on the technique used for administration; local nasal cavity factors such as swelled nasal mucosa with diminished air exchange in the nostril; local factors at the nasal mucosa such as increased amount of mucus.

Patient # 6 scored 3–3 and hence was not in great distress before treatment. In some cases the anesthesia nurse tries to foresee a possible need for analgesia in conjunction with helicopter loading or realignment of a fracture and hence administers analgesics to a patient in no apparent present distress. This may have been the case with patient # 6.

S-ketamine exerts its actions in the brain and if the effect from nasal administration is via a direct olfactory mucosal uptake to the brain or from the systemic effect from an uptake in the blood and then via the bloodstream to the brain is not clear. It is clear though from a study by Weber *et al.* that clinically relevant concentrations in the blood are accomplished within minutes of a nasal administration [6]. The onset of analgesia (3–10 minutes in our experience) suggests that the effect comes via the blood stream.

In accordance with the results of Malinovsky et al. [5], some patients had a rather slow analgesic onset. This may depend on a variable amount of the drug deposited on the nasal mucosa and a variable amount swallowed which in turn may depend on local conditions at the nasal mucosa or mishaps at administration (sneezing or coughing). We also believe that the direction that one sprays the drug is important. If the spray hits a solid object early on, for example the nasal partition wall, it will all condense to a liquid form and run either down to pharynx or out of the nostril, depending on the position of the head. It is more likely to have a successful administration if the patient cooperates and inhales through the nostril in question and not to administer more than 0,5-1,0 ml at a time (depending on patient size).

S-ketamine was developed to decrease psychotomimetic side-effects of racemic ketamine but a review suggests that this is not clearly the case [7]. Another side-effect of ketamine as well as S-ketamine is hypersecretion. Neither of these complications was reported in our patients, nor nausea although three of them experienced vertigo. Many complained over the taste of the medication and we recommend warning on beforehand, especially when treating children. S-ketamine potency is twice that of racemic ketamine, hence equal volumes of the drug would in theory have the same biological effect since S-ketamine is accessible in 25 mg/ml and ketamine in 50 mg/ml.

S-ketamine is, as opposed to ketamine, free of the preservatives that have been linked to neural toxicity [8,9]. This is why S-ketamine is used for epidural anaesthesia and ketamine not. Since there may be a direct uptake from the nasal mucosa via the olfactory lobe to the central nervous system, there have been recommendations not to use ketamine for nasal administration either. The issues of the potential neurotoxicity is still under debate and while Vranken et al. found a neurotoxic effect of daily intratecal administration for one week of preservative free S-ketamine in rabbits, Rojas et al. showed that a single epidural injection of 1 mg/kg S-ketamine did not produce neural lesions in dogs [10].

We found that patients always responded to verbal stimuli but some were not adequate in their response. We had no impression of compromised airway or airway reflexes.

To summarize, the opinion of our experienced prehospital staff was that nasal S-ketamine offered a fast, easy, and essentially non-invasive way of reducing acute pain secondary to trauma, without appreciable side effects.

Further advantages are a relatively low cost of the treatment, a needleless approach that diminish the risk of transmission of blood-borne infections, and supposedly a reduced rescue-time which is particularly valuable in a cold or otherwise dangerous environment. All together these benefits make it an appealing strategy for prehospital service in general and even more so in developing countries. The main reason why we only use it as a last resource strategy is that it is off label use of the drug. If our impression of a safe and relatively rapid analgesic effect of nasal S-ketamine could be confirmed, it opens for a widening of its formal indications which would facilitate improved prehospital pain therapy.

The limitation of our data is first of all that it is not a study but only a series of nine case-reports and as such not suitable for any generalisation. There is no control-group and there is a possible bias in that the decision to use the nasal strategy was done on the scene.

We suggest that the feasibility of nasal S-ketamine for prehospital analgesia should be evaluated in a formal randomised controlled trial. Such a trial would be hard to design as blinded but may as well have an open design. It might look into questions such as the safety of the treatment, the effect of nasal analgesia compared to standard care, onset time, the frequency and type of side-effects and the necessary rescue-time spent on the scene of accident.

## Conclusion

We conclude that nasal administration of S-ketamine for treatment of traumatic pain on the scene of an accident seems to be a promising alternative in cases where vascular access is foreseen or proven problematic.

## Competing interests

The authors declare that they have no conflicts of interests.

## Authors’ contributions

JJ, JS, ES and HZ conceived the idea to test nasal S-ketamine for challenging cases and decided to do a follow up on the efficacy of treatment. JJ wrote the manuscript. JS helped designing the prehospital pain therapy protocol for challenging cases. MN included patients and has helped preparing the manuscript as well as refining the pain therapy protocol. FS helped with preparation of the manuscript and interpretation of data. HZ revised the manuscript. All authors read and approved the final manuscript.
